# Action of Spirits upon Habitual Drunkards

**Published:** 1842-03

**Authors:** C. H. Schultz

**Affiliations:** Berlin


### Selections from American and Foreign Journals.

---

*Action of Spirits upon Habitual Drunkards.* By Prof. C. H. Schultz, of Berlin. There are two points into which this subject naturally divides itself:—1*st*, What is the nature of the action of ardent spirits on habitual drunkards? 2*d*, Why does not wine produce, equally with spirits, those morbid effects which are observed in the habitual drunkard?

Professor Schultz distinguishes between intoxication—a merely temporary effect of ardent spirits, the result of their physiological and medical action in excess—and their pathological action, which shows itself in its highest degree in the production of delirium tremens. Intoxication is a brief excitement, and one, to a certain extent, normal, which ceases when the remote cause is removed; but tremors and delirium are the result of a morbid reaction, which continues for a long time after the cause has ceased to act. In investigating the nature of the morbid condition to which habitual spirit drinking gives rise, we must take no account of its short stimulating effect, which is rather the opposite of the disease produced by spirits, than its first stage.

Some writers have regarded this disease as an over-excitement of the nervous system, amounting almost to an inflammatory condition, but ending in exhaustion. Others have considered it to be the result of the direct action of spirits on the blood, and have regarded the nervous symptoms merely as secondary. The dark color of the blood of drunkards, which several persons have noticed, favors the supposition, that some special change is produced in that fluid. The causes, however, by which the color of the blood may be modified are so various, that a mere knowledge of the fact that such a change occurs, is not enough to solve any of the difficulties of the subject. The supposition that this change consists in an excess of hydrogen and carbon in the blood, is contradicted by the dissimilarity of the symptoms which intoxication produces, from those which result from respiring any of the narcotic gases. The analogy between the states of the blood under those two conditions is merely apparent, and has not been shown to exist in any point besides color. Orfila and Renard have attached much importance to the chemical action of spirits on the mucous membrane of the intestines; alcohol coagulating albumen, but having in other respects an antiseptic property. It has been supposed, by the means of this property, to interfere with digestion; but this opinon is in many respects erroneous, for there is a great tendency to decomposition in the contents of the intestinal canal of habitual spirit drinkers.

Dr. Schultz is of opinion, that the primary action of ardent spirits is upon the organs of vegetative life. Two points connected with the subject have especially attracted his attention:—1*st*, The influence of ardent spirits on the bile, and its consequences. 2*d*, Their influence on the coloring matter of the blood, and on the envelope of the blood corpuscles.

If alcohol is added to bile, and the solution evaporated for a short time, the bile loses its alkaline reaction; it likewise ceases to be precipitated by vinegar, dilute sulphuric or muriatic acids, or by solutions of oxalic or phosphoric acids. The sour contents of the stomach of rabbits, dogs, and oxen, throw down no precipitate from the bile of oxen, when mixed with alcohol, and a long time is requisite for neutralizing the acid, while sometimes that change does not take place at all, if the bile and accohol have been long mixed together. This fact throws a new light on the disordered digestion of drunkards, and especially upon the generation of acid in the stomach, and the sour eructations to which they are so especially liable. But the bile is not only directly subservient to digestion, for it contains besides a number of excrementitious matters. In the healthy state these matters are got rid of by being precipitated in insoluble flocculi, which are then voided with the feces. The alcoholic solution of bile is not precipitated by acids, or by the contents of the stomach, or but very imperfectly, and consequently these excrementitious matters are retained in a state of solution in the intestinal canal, and become mixed with the chyle. The occurrence of jaundice, and many of the icteric symptoms to which drunkards are liable, may be partly explained, by supposing some of this morbid bile to become absorbed, and to enter the circulation.

It has long been known that a great part of the ardent spirits taken into the stomach, is absorbed unchanged, and that alcohol thus finds its way into the blood. Hitherto, however, persons have contented themselves with saying, that the blood has more of the characters of venous blood than natural, and they have not inquired which of the constituents of the fluid undergoes the morbid change. Professor Schultz has made the following experiments:—If a small quantity of spirits of wine is added to fresh blood, the fluid becomes transparent, and its natural color changes to a cherry red, but it does not become blacker, as is commonly asserted. If the blood is now examined under the microscope, it will be seen that the coloring matter generally is changing its situation, and, instead of being contained in the blood corpuscles, becomes gradually diffused through the plasina. Thus, (if the alcohol is added to blood deprived of its fibrine,) in the course of a short time, instead of colored corpuscles floating in a colorless plasina, the red plasina will be seen to contain colorless corpuscles. If the spirits are added to fresh blood capable of coagulating, the red plasina will form into a gelatiniform substance of the consistence of thick milk, and no separation into crassamentum and serum will afterwards take place. If half the quantity of spirit is added to the blood, coagulation of the plasina, or of the albumen of the serum, in cases where the blood has been deprived of its serum, at once takes place, and forms a mass of the consistence of cheese. These alterations are produced by injecting spirit into the veins of living animals, as well as by adding it to blood out of the body, and instant death of the animal takes place, although the change of the blood does not extend beyond that part of the venous system into which the injection is thrown. The changes in the capsule of the blood globules likewise merit attention. They lose their color gradually, and contract more or less in size according to the quantity of alcohol added. In some instances the blood corpuscles contract almost to a point, and become nearly undistinguishable, so that the blood appears to be a uniformly-transparent red fluid, without any globules. The newly-formed corpuscles undergo these changes most rapidly, while the alterations take place much more slowly in the larger globules, which contain more coloring matter.

The changes which the blood undergoes, are not directly chemical, for alcohol does not dissolve the coloring matter, but the corpuscles seem to lose it owing to their contraction. The constituents of the blood, then, are not actually decomposed, as was formerly imagined; but the results of the action of alcohol are not on that account less pernicious. The process of restoration is dependent on the normal condition of the blood corpuscles, since they are the media through which the mutual changes in the air and blood are effected. The state of the coloring matter is also a point of great importance, as is evident from the striking changes which the air produces, and which are so marked that we look on the alteration of color as the chief point of difference between venous and arterial blood. But both the contractility of the blood globules and their contents, are greatly modified by the action of spirits on the blood. Thus we arrive at an explanation of the alterations in the respiratory process of the habitual spirit drinkers, which result principally from the proper changes not taking place in the blood corpuscles and their contents. Less oxygen than natural is absorbed, hence less carbonic acid is exhaled, and the blood assumes a venous character, though one widely differing from that which the narcotic gases produce, since they produce a dilatation of the blood globules, and an accumulation of coloring matter within them.

From these alterations Professor Schultz deduces the various morbid conditions of the nutritive process incidental to spirit drinkers. The action of spirit on the nervous system is usually physiological rather than pathological. Delirium tremens indeed is the result of a pathological process; not however of exhaustion consequent on over-irritation of the brain and nerves, but rather of a destruction of their excitability, owing to the morbid changes in the blood.—*Monthly Jour. Med, Sci.* Oct. 1841, from *Hufeland's Journal*, Apr. 1841.

